# Working re-organisation due to the pandemic may negatively affect workers’ quality of life

**DOI:** 10.1093/eurpub/ckac131.273

**Published:** 2022-10-25

**Authors:** F Ghelli, V Bellisario, G Squillacioti, M Panizzolo, R Bono

**Affiliations:** Department of Public Health and Pediatrics, Università degli Studi di Torino, Turin, Italy

## Abstract

**Background:**

From the beginning of 2020, the COVID-19 pandemic imposed many changes in the organization of our personal and professional life, allowing a shift to teleworking and hybrid working arrangements worldwide. The persistence of this condition determined the integration of these changes in the daily routine, and our aim was to investigate how these changes could affect workers’ Quality of Life (QOL) in the long run.

**Methods:**

An online questionnaire was administered to 650 workers employed in an Italian company in July 2021. The domains considered were socio-demographic characteristics, QOL (WHOQOL-bref and Healthy Days), lifestyle, health status, physical activity, and work-related factors. Comparisons were performed with non-parametric tests.

**Results:**

Among the 332 respondents (response rate = 51.1%), 79.2% were white-collar workers. The concern for the pandemic affected workers’ QOL, especially in the physical health and the environmental domains (p < 0.001 and p = 0.001, respectively), with lower values in subjects reporting the highest values of concern. A similar effect was found also for healthy days concerning physical and psychological health, and the ability to perform usual activities (p = 0.003, p = 0.003, and p = 0.029, respectively). As well, changes in working arrangements significantly affected the environmental and physical domains (p = 0.023 and p = 0.015, respectively) and the ability to do usual activities (p = 0.011), with lower values in those who interrupted the working activity. Workers whose activity required a shift to teleworking reported higher scores in the physical health domain (p = 0.041) and a higher number of days with good physical health (p = 0.002), while a lower number of days with good psychological health (p = 0.006).

**Conclusions:**

These preliminary data revealed that organizational strategies adopted in working scenarios to contain the spread of COVID-19 may have an impact on workers’ QOL, as well as the concern for the pandemic.

**Key messages:**

• Changes in the working arrangements due to pandemic may negatively affect the workers’ Quality of Life, especially for those who had to interrupt their working activity.

• The shift to telework, even if appearing to be beneficial for physical health perception, seems to negatively affect workers’ psychological health.

